# PCR-fingerprinting of culturable yeasts from commercially obtained beers: a simple and engaging applied microbiological laboratory exercise

**DOI:** 10.17912/micropub.biology.001381

**Published:** 2025-02-19

**Authors:** Walter P. Pfliegler, Alexandra Imre, University of Debrecen Biotechnology BSc Class of 2025, István Pócsi

**Affiliations:** 1 Molecular Biotechnology and Microbiology, University of Debrecen, Debrecen, Hajdú-Bihar, Hungary; 2 University of Debrecen, Debrecen, Hajdú-Bihar, Hungary

## Abstract

Yeast and fermented products present an opportunity to introduce students to applied microbiology. We designed and implemented a project-oriented laboratory class where yeasts from bottled beverages were isolated and compared using DNA fingerprinting and Sanger-sequencing. We recovered 17 *Saccharomyces* isolates, and two non- *Saccharomyces* yeasts. Fingerprinting identified two groups of closely related *Saccharomyces* isolates in unrelated beer styles, later identified as diastatic and wine yeasts using phylogenomics. Isolates from traditional products thus may not represent the original fermentation. We believe that the interlinked nature of topics and the simple basis can elevate engagement and performance of students during such a class.

**Figure 1. Genotyping results of the isolated Saccharomyces yeasts:**
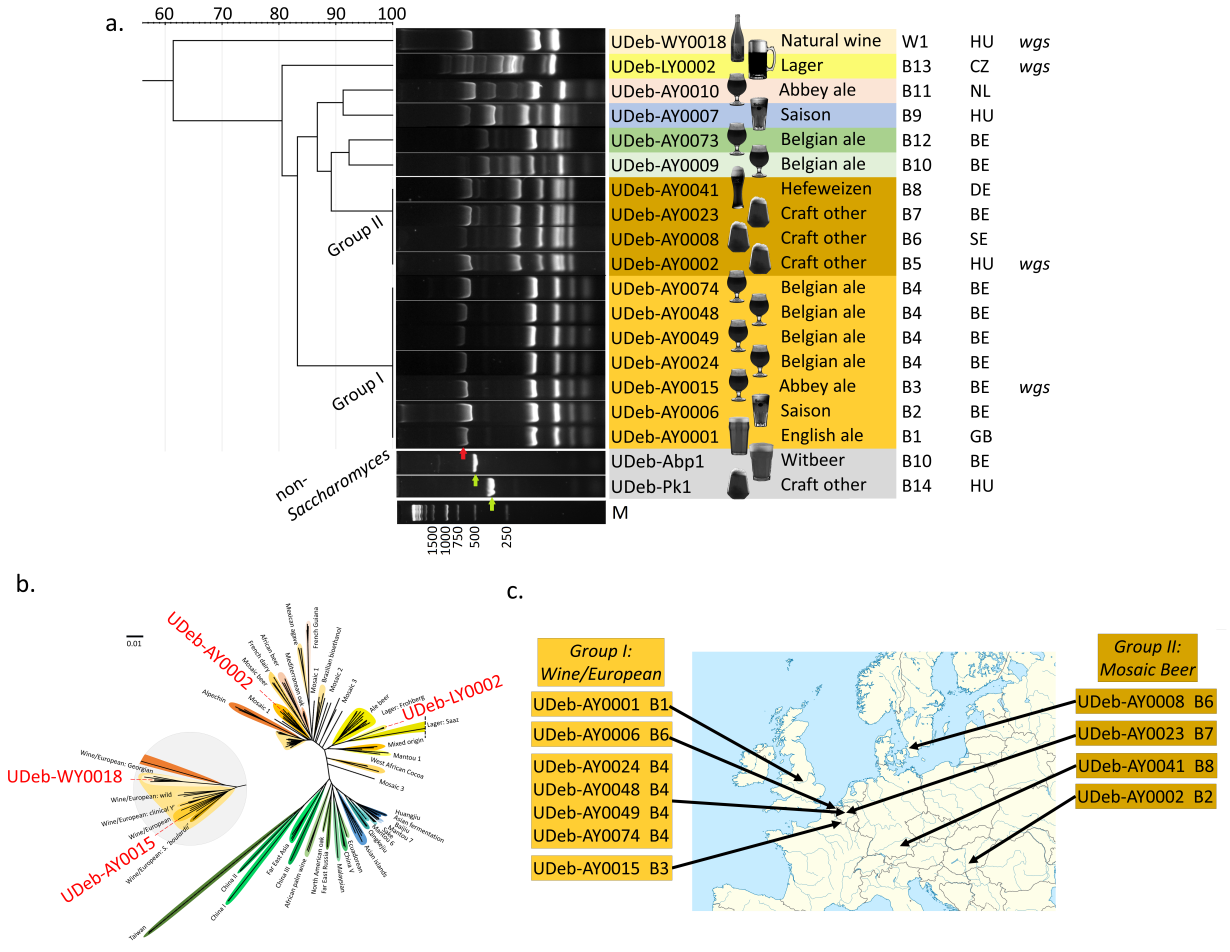
**a.** UPGMA dendrogram based on multiplex fingerprinting results obtained for *Saccharomyces* isolates analyzed in this study. Two groups of isolates, with identical band patterns in each, are marked as Group I and II and with colored background. Other band patterns are delineated by various background colors. Type of the product of origin is indicated with pictograms and short descriptions, breweries and the sole winery of origin are indicated with their identifiers (B=brewery, W=winery) in the next column, and country codes represent origin of the products (BE, Belgium; CZ, Czechia; DE, Germany; GB, Great Britain; HU, Hungary; NL, Netherlands; SE, Sweden). In the last column, ‘wgs’ indicates isolates with sequenced genomes. Control ITS bands obtained for non- *Saccharomyces* yeasts are also shown for comparison. The ITS bands are marked for non- *Saccharomyces* yeasts with green and for *Saccharomyces* with red arrowheads. Lane marked with ’M’ shows 1 kb size marker, bands are marked for their length. **b.** Phylogenomic analysis of four selected isolates. *S. cerevisiae* clades and lager yeasts are included from the literature, and the isolates sequenced here are marked in red text. Clades are indicated by colored backgrounds. The long branch of the ‘Lager: Saaz’ clade is shortened for visibility purposes. **c.** Brewery origin of the yeasts from the two groups of *Saccharomyces* with identical band patterns in various countries in Europe.

## Description

The budding yeast *Saccharomyces cerevisiae* is one of the most widely known and used microbes. Recently, its global phylogenomic diversity has been uncovered to an unprecedented scale (Peter et al. 2018; Pontes et al. 2020) . Two main clades of the species have adapted for ale beer brewing, namely the Ale 1 and the Mosaic (diastatic) beer (Gallone et al. 2018; Peter et al. 2018; Paraíso et al. 2023) . The Ale 1 yeasts have several described subgroups, while the Mosaic beer clade includes diastatic strains (Krogerus and Gibson 2020) . A third group of traditional farmhouse ale yeasts has recently been described (Preiss et al. 2018; 2024) . In bottom-fermented lager beer production, the Saaz or the Frohberg type of *S. pastorianus* , a hybrid of *S. cerevisiae* and *S. eubayanus* is used, and several other hybrids have also been described (Langdon et al. 2019; Gallone et al. 2019) .

Beer, through much of its history, still contained live yeasts following fermentation and was stored in wooden barrels and casks where a sufficient residual extract allowed for refermentation (Flavin et al. 2023; Štulíková et al. 2020), leading to carbonation. Since the end of the 19 ^th^ century, bottled beers became prevalent, followed by many technological improvements, more standardized and eventually most often pasteurized products (Raihofer et al. 2022) . Traditional abbey ales, along with some specialty products such as lambic beers, have retained the method of bottle refermentation (Derdelinckx et al. 1995) . Unfiltered and unpasteurized beers have also retained a share in the market. Currently, the emerging craft beer scene and customer demand for innovation led to bottle or cask refermentation to be more widely used once again (Štulíková et al. 2020; Marconi et al. 2016; Baiano 2021). In addition, live yeasts may also be introduced to bottled products inadvertently (Meier-Dörnberg et al. 2018; Krogerus and Gibson 2020) . Other traditional fermented beverages have experienced a craft revival, too, e.g. natural and unfiltered wines and pét-nats (Wei et al. 2023) .

Scientific and industry interest in locally selected beer strains increased recently (Bonatto 2021; Kerruish et al. 2024) . Isolates both from primary fermentation and from bottled beer have been sequenced by various research groups, and the home brewing community has also seen more and more interest in isolates sourced from bottles.

Here, we describe a simple set of experiments aimed at raising student engagement in brewing microbiology that revolves around the topic of beer bottle isolates. The experiments were successfully applied in the BSc-level Biotechnology program at the Faculty of Science and Technology of the University of Debrecen. Inspired by the BREWMOR initiative (Bridging Research and Education with Model Organisms – www.brewmor.org) a network of teaching and research faculty dedicated to propagating experiential learning for biology students, and by published student projects such as the yEvo (Moresi et al. 2024) our Department organized two consecutive single-semester courses for students designed to (1) teach scientific literature and database search and analysis of biomedical patents, and then to (2) teach the isolation and analysis of yeasts with the purpose of assessing *Saccharomyces* diversity in bottled beverages.

During the first (seminar-type) class, database searches, scientometrics, and the basics of intellectual property in science were covered. Topics included phylogenomic studies on beer yeasts, innovations in brewing, the largest producers of yeast starters, and patents (e.g. Farber, 2019) of the companies. Students prepared project works on the topics and discussed trends, marketing strategies in brewing, and legal perspectives – specifically, the Nagoya protocol (European Commission, 2021) . Details on topics are listed in a supplementary document (doi: 10.6084/m9.figshare.28407530). Based on experience during the class, we found that student engagement was helped by the interlinked nature of topics.

In the second semester of first-year students, a “General and Applied Microbiology” lab course was organized where students learnt how to isolate pure cultures of yeasts from the sediment of commercially obtained beer bottles of various origin, along with a single natural wine sample, all purchased locally (Table 1). Multiplex PCR-fingerprinting analysis (as described in Imre et al. 2019) was applied to the isolates, to compare *Saccharomyces* samples (Table 2). The method includes a control ITS primer pair, resulting in a control band for fungi (around 750 bp for *Saccharomyces* but markedly shorter in most other yeasts), *S. cerevisiae* -specific microsatellite primer pairs, and primers specific to *S. cerevisiae* delta sequences. Thus, this fingerprinting results in a single ITS band in the case of non- *Saccharomyces* samples, and multiple bands for *S. cerevisiae* ( Figure 1 a).

Two of the isolates only showed the single ITS control ( Figure 1 a) and were identified as *Aureobasidium pullulans* and *Pichia kluyveri* subsequently (Table 1). A further 17 isolates (Table 1) showed *Saccharomyces* band patterns ( Figure 1 a). Bands were scored and compared, and students were asked to interpret the results. Students successfully completing the course were included as group authors in this publication.

As shown in Figure 1 a, two groups, consisting of seven and four isolates, were identified with identical band patterns, indicating strain-level identity or very close relatedness. The first (Fingerprinting Group I) consisted of six Belgian ale isolates and a single UK isolate, all from bottle conditioned/refermented beers. Four of the seven isolates originated from a single brewery’s products ( Figure 1 a,c, Table 1). The yeasts collected from these may represent a commonly used strain or closely related group of strains, or alternatively, a common contaminant. The second group’s samples (Fingerprinting Group II) are from products with no statement on bottle refermentation. They were found in beer styles normally associated with their specific yeasts (IPA, pilsner IPA, kveik ale, Weißbier/Hefeweizen) ( Figure 1 a,c; Table 1). It is thus highly unlikely that the yeasts strain isolated from them was responsible for primary fermentation; it is plausibly a common contaminant strain. The remaining yeasts displayed unique patterns, with the most different being the Czech lager and natural wine isolates ( Figure 1 a).

Students were tasked to interpret the results and discuss them in their lab notes. For the discussion, the publications on beer yeast diversity and the clades used during the previous seminar proved useful. Students also had to discuss how a more definitive subtyping of the isolates could be achieved to identify the clades the yeasts belong to. The possible approach, phylogenomics, was shortly discussed in the light of the above-mentioned earlier studies.

Although phylogenomics was out of the scope of the class, we later chose four isolates for sequencing and a phylogenomics ( Figure 1 b). Based on coverage data, only the isolate from the unfiltered unpasteurized Czech lager, UDeb-LY0002, proved to be a hybrid. Its *S. cerevisiae* subgenome was grouped into the Lager: Frohberg clade (coverage graph doi: 10.6084/m9.figshare.27988580). To our knowledge, the survival of lager yeasts after bottling has not been shown using molecular genetic data previously. The phylogenomic dendrogram also showed that the natural wine isolate UDeb-WY0018 belonged to the Wine/European – Georgian subclade, while the UDeb-AY0002 isolate, representing the Fingerprinting Group II, was a member of the diastatic Mosaic Beer clade. The ale isolate UDeb-BY0015 from the Fingerprinting Group I, however, belonged to the Wine/European clade and might represent a bottle conditioning yeast ( Figure 1 b).

Our results highlight that care must be taken when bottle isolates are examined in context of traditional beer styles and yeast clades as they may not be the original strains responsible for primary fermentation. Breweries rarely publish the identities of their strains and bottle refermentation may or may not involve the use of specific commercially available bottle conditioning yeasts (such as Fermentis Safbrew T-58, S-33, or LalBrew CBC-1, a repurposed Champagne yeast) (Štulíková et al. 2020). Diastatic yeast contaminants may further complicate the picture (Paraíso et al. 2023) . Other species may also be found in bottled beer. These aspects all present multi-faceted and engaging opportunities to be discussed with students according to our experience, as they encounter a gradually unfolding complexity of industrial microbes. Costs associated with such a beer-yeast focused laboratory class are also manageable. Genome sequencing and bioinformatics may enhance the findings subsequently, as shown here.

Further studies may extend the scope of bottled products. For example, lambic beers are known to have a diverse microbiota which is only known to the level of species, not clades (Bongaerts et al. 2024) , providing opportunities for more studies especially in the case of bottled products that harbor live microbes easily accessible to consumers.

## Methods

**Yeast isolation.** Beer and natural wine samples were commercially obtained in Hungary for the purpose of isolation. After decanting the product, sediments were suspended in sterile YPD (yeast extract, peptone, dextrose, VWR Chemicals, Solon, OH, USA) and spread to YPD agar plates and incubated for 2–3 days at 22°C. Colonies were further purified with sterile inoculation loops until single-cell derived colonies were obtained. Isolates were deposited into our culture collection at –70°C in YPD medium supplemented with 30% v/v glycerol.

**Yeast identification.** Colony DNA for PCR tests was isolated according to Lõoke et al. (Lõoke, Kristjuhan, and Kristjuhan 2011) from the colonies and stored in 1×TE. Briefly, a part of yeast colony was suspended in 100 µl lysis buffer (200 mM LiOAc 1% SDS solution), and incubated at 70°C for 15 min, then 300 μl 96% ethanol was added, the samples were mixed by brief vortexing. DNA was collected by centrifugation at 4°C with 15,000× g for 10 min. The precipitate was dissolved in 100 μl TE, then cell debris was spun down by brief centrifugation (15,000× g for 1 min), and 1 μl supernatant was used for a PCR reaction of 50 μl end volume. These colony DNA samples were used to differentiate *Saccharomyces* samples from other yeasts. We used the interdelta and microsatellite fingerprinting multiplex PCR combining interdelta, microsatellite ( *YLR177w* , *YOR267c* ), and as a control, ITS 1–4 primer pairs (Imre et al. 2019) with the GoTaq G2 polymerase (Promega, Madison, WI, USA) (Tables 2,3). We performed gel electrophoresis of the products (2% TBE agarose, 90 min, 100 V), Gene Ruler 1 kb size marker (Thermo Fischer Scientific, Waltham, MA, USA) was used for each run. We identified *Saccharomyces* samples based on band patterns, the non- *Saccharomyces* yeasts were subjected to PCR targeting the 26S ribosomal large subunit rRNA gene. The GoTaq G2 polymerase was used with primers NL1 and NL4 (Tables 2,3). The PCR products were subjected to capillary sequencing after PCR cleanup with the E.Z.N.A. cycle pure kit (Omega Bio-Tek, Norcross, GA, USA) by the sequencing core facility of the University of Debrecen. Sequenograms were manually inspected and the NCBI BLAST service was used for species identification, whereby the species with the closest hit in the NCBI GenBank was considered as a putative species identification, and then the sequence of the hit species’ type strain was once again aligned to the query sequence using BLAST. A similarity of >99% with the type was considered a definitive species identification. Sequences were deposited in GenBank (accession numbers: UDeb-Abp1: PQ381248; UDeb-Pk1: PQ381247).

**Fingerprinting analysis.** The *Saccharomyces* isolates identified as described above, were subjected to another round of multiplex PCR and gel electrophoresis for fingerprinting. In this case, genomic DNA was isolated using the glass bead method as described in Hanna and Xiao (2006). Concentration was set to 100 ng/µl in TE buffer. This better-quality genomic DNA is adviseable for a more reproducible and clearer fingerprinting band pattern. Multiplex fingerprinting was performed (Imre et al. 2019) with the GoTaq Flexi Hot Start polymerase (Table 2), gel electrophoresis conditions were the same as described above (Table 3). Band patterns were analyzed using GelJ (Heras et al. 2015) implementing the UPGMA clustering method with Dice coefficient. Original gel photos are uploaded to FigShare (doi: 10.6084/m9.figshare.28365200).

**Whole genome sequencing and phylogenomics.** We chose four isolates to be subjected to whole-genome sequencing using Illumina technology and a phylogenomic analysis involving members of previously described clades was performed after mapping to a concatenated reference of the species of the *Saccharomyces* genus. Genomes used are detailed in a supplementary table at FigShare (doi: 10.6084/m9.figshare.27988601). Mapping was followed by allele calling and generating a neighbor-joining tree as described in detail in the supplementary methods (doi: 10.6084/m9.figshare.27988613). The NEWICK file of the dendrogram was uploaded to FigShare (doi: 10.6084/m9.figshare.27988586). Coverage was visualized for the species of the genus from the mapping .bam files in bins of 10kb with a sliding window approach and this was used to check whether any of the isolates were hybrids. Raw sequencing reads were deposited in BioProject PRJNA1195563 of the NCBI Sequence Read Archive.

## Reagents

**Table 1.** Yeast isolates analyzed in this study.

**Table:** 

**Fingerprinting group**	**Strain**	**Species**	**Isolation source**	**Manufacturer’s disclosure about yeasts in product**	**Origin of product**
I	UDeb-AY0001	*S. cerevisiae*	Brewery 1: Amber ale	bottle conditioned	UK
I	UDeb-AY0006	*S. cerevisiae*	Brewery 2: Saison	bottle refermentation	Belgium
I	UDeb-AY0015	*S. cerevisiae*	Brewery 3: Belgian abbey ale	bottle refermentation	Belgium
I	UDeb-AY0024	*S. cerevisiae*	Brewery 4: Belgian ale	bottle refermentation	Belgium
I	UDeb-AY0048	*S. cerevisiae*	Brewery 4: Belgian tripel ale	bottle refermentation	Belgium
I	UDeb-AY0049	*S. cerevisiae*	Brewery 4: Belgian tripel ale	bottle refermentation	Belgium
I	UDeb-AY0074	*S. cerevisiae*	Brewery 4: Belgian ale	bottle refermentation	Belgium
II	UDeb-AY0002	*S. cerevisiae*	Brewery 5: IPA	pilsner yeast used for primary fermentation	Hungary
II	UDeb-AY0008	*S. cerevisiae*	Brewery 6: kveik pale ale	kveik yeast used for primary fermentation	Sweden
II	UDeb-AY0023	*S. cerevisiae*	Brewery 7: dark IPA	none	Belgium
II	UDeb-AY0041	*S. cerevisiae*	Brewery 8: dark Hefeweizen	unfiltered	Germany
	UDeb-AY0007	*S. cerevisiae*	Brewery 9: Saison	saison used for primary fermentation	Hungary
	UDeb-AY0009	*S. cerevisiae*	Brewery 10: Belgian ale	bottle refermentation	Belgium
	UDeb-AY0010	*S. cerevisiae*	Brewery 11: Dutch abbey quadrupel	bottle refermentation	Netherlands
	UDeb-AY0073	*S. cerevisiae*	Brewery 12: Belgian ale	bottle conditioned	Belgium
	UDeb-LY0002	*S. pastorianus*	Brewery 13: Lager	unpasteurized, unfiltered	Czechia
	UDeb-WY0018	*S. cerevisiae*	Winery 1: natural white wine	spontaneosly fermented, unfiltered	Hungary
	UDeb-Abp1	*Aureobasidium pullulans*	Brewery 10: Belgian witbier	none	Belgium
	UDeb-Pk1	*Pichia kluyveri*	Brewery 14: amber ale	none	Hungary

**Table 2.** Primers used in this study

**Table:** 

**Purpose**	**Primer name**	**Primer sequence**
Multiplex fingerprinting	δ12	TCAACAATGGAATCCCAAC
Multiplex fingerprinting	δ2	GTGGATTTTTATTCCAACA
Multiplex fingerprinting	YLR177wf	CTTAAACAACAGCTCCCAAA
Multiplex fingerprinting	YLR177wr	ATGAATCAGCGCATCAGAAAT
Multiplex fingerprinting	YOR267cf	ATGACTGCAGCAATGAATCG
Multiplex fingerprinting	YOR267cr	TCCTCTGTGCTGTTGACTCG
Multiplex fingerprinting	ITS1	TCCGTAGGTGAACCTGCGG
Multiplex fingerprinting	ITS4	TCCTCCGCTTATTGATATGC
Species identification	NL1	GCATATCAATAAGCGGAGGAAAAG
Species identification	NL4	GGTCCGTGTTTCAAGACGG

**Table 3.** PCR program used in this study.

**Table:** 

Experiment	PCR mix for a 50 μl reaction	Program
Multiplex fingerprinting	1× GoTaq Flexi Buffer; 4.0 mM MgCl _2_ ; 0.2 mM each dNTP; 20 pmol of primers δ12, δ2, YLR177wf, YLR177wr each; 10 pmol of primers YOR267cf, YOR267cr each; 3 pmol of primers ITS1, ITS4 each; 2.5 u GoTaq G2 Polymerase; 1 ng genomic DNA	95 °C 3 min, 25× (95 °C 30 sec, 55 °C 30 sec, 72 °C 1 min), 72 °C 5 min
Species identification	1× GoTaq Flexi Buffer; 4.0 mM MgCl _2_ ; 0.2 mM each dNTP; 20 pmol of primers NL1, NL4; 2.5 u GoTaq G2 Polymerase; 1 ng genomic DNA	95 °C 3 min, 30× (95 °C 30 sec, 60 °C 30 sec, 72 °C 1 min), 72 °C 5 min

## References

[R1] Baiano A (2020). Craft beer: An overview.. Compr Rev Food Sci Food Saf.

[R2] Bonatto D (2021). The diversity of commercially available ale and lager yeast strains and the impact of brewer's preferential yeast choice on the fermentative beer profiles.. Food Res Int.

[R3] Bongaerts D, Bouchez A, De Roos J, Cnockaert M, Wieme AD, Vandamme P, Weckx S, De Vuyst L (2024). Refermentation and maturation of lambic beer in bottles: a necessary step for gueuze production.. Appl Environ Microbiol.

[R4] Derdelinckx GH, Neven H, Arnott P, Demeyer I, Delvaux F. 1995. Belgian special beers: Refermented beers; white and wheat beers; amber and dark beers; spiced and hoppy beers. Cerevisia Biotechnol. 20: 67–73.

[R5] European Commission. 2021. Guidance document on the scope of application and core obligations of Regulation (EU) No 511/2014 of the European Parliament and of the Council on the compliance measures for users from the Nagoya Protocol on Access to Genetic Resources and the Fair and Equitable Sharing of Benefits Arising from their Utilisation in the Union. Official Journal of the European Union C 13/1.

[R6] Farber MJ. 2019. WO 2019/018803 A1 Compositions and methods for brewing sour beer.

[R7] Flavin Susan, Meltonville Marc, Taverner Charlie, Reid Joshua, Lawrence Stephen, Belloch-Molina Carlos, Morrissey John (2023). Understanding Early Modern Beer: An Interdisciplinary Case-Study. The Historical Journal.

[R8] Gallone B, Mertens S, Gordon JL, Maere S, Verstrepen KJ, Steensels J (2017). Origins, evolution, domestication and diversity of Saccharomyces beer yeasts.. Curr Opin Biotechnol.

[R9] Gallone B, Steensels J, Mertens S, Dzialo MC, Gordon JL, Wauters R, Theßeling FA, Bellinazzo F, Saels V, Herrera-Malaver B, Prahl T, White C, Hutzler M, Meußdoerffer F, Malcorps P, Souffriau B, Daenen L, Baele G, Maere S, Verstrepen KJ (2019). Interspecific hybridization facilitates niche adaptation in beer yeast.. Nat Ecol Evol.

[R10] Hanna Michelle, Xiao Wei (2013). Isolation of Nucleic Acids. Yeast Protocols.

[R11] Heras J, Domínguez C, Mata E, Pascual V, Lozano C, Torres C, Zarazaga M (2015). GelJ--a tool for analyzing DNA fingerprint gel images.. BMC Bioinformatics.

[R12] Imre A, Rácz HV, Antunovics Z, Rádai Z, Kovács R, Lopandic K, Pócsi I, Pfliegler WP (2019). A new, rapid multiplex PCR method identifies frequent probiotic origin among clinical Saccharomyces isolates.. Microbiol Res.

[R13] Kerruish DWM, Cormican P, Kenny EM, Kearns J, Colgan E, Boulton CA, Stelma SNE (2024). The origins of the Guinness stout yeast.. Commun Biol.

[R14] Krogerus K, Gibson B (2020). A re-evaluation of diastatic Saccharomyces cerevisiae strains and their role in brewing.. Appl Microbiol Biotechnol.

[R15] Langdon QK, Peris D, Baker EP, Opulente DA, Nguyen HV, Bond U, Gonçalves P, Sampaio JP, Libkind D, Hittinger CT (2019). Fermentation innovation through complex hybridization of wild and domesticated yeasts.. Nat Ecol Evol.

[R16] Lõoke M, Kristjuhan K, Kristjuhan A (2011). Extraction of genomic DNA from yeasts for PCR-based applications.. Biotechniques.

[R17] Marconi O, Rossi S, Galgano F, Sileoni V, Perretti G (2016). Influence of yeast strain, priming solution and temperature on beer bottle conditioning.. J Sci Food Agric.

[R18] Meier-Dörnberg T, Kory OI, Jacob F, Michel M, Hutzler M (2018). Saccharomyces cerevisiae variety diastaticus friend or foe?-spoilage potential and brewing ability of different Saccharomyces cerevisiae variety diastaticus yeast isolates by genetic, phenotypic and physiological characterization.. FEMS Yeast Res.

[R19] Moresi NG, Geck RC, Skophammer R, Godin D, Students Y, Taylor MB, Dunham MJ (2023). Caffeine-tolerant mutations selected through an at-home yeast experimental evolution teaching lab.. MicroPubl Biol.

[R20] Paraíso F, Pontes A, Neves J, Lebani K, Hutzler M, Zhou N, Sampaio JP (2023). Do microbes evade domestication? - Evaluating potential ferality among diastatic Saccharomyces cerevisiae.. Food Microbiol.

[R21] Peter J, De Chiara M, Friedrich A, Yue JX, Pflieger D, Bergström A, Sigwalt A, Barre B, Freel K, Llored A, Cruaud C, Labadie K, Aury JM, Istace B, Lebrigand K, Barbry P, Engelen S, Lemainque A, Wincker P, Liti G, Schacherer J (2018). Genome evolution across 1,011 Saccharomyces cerevisiae isolates.. Nature.

[R22] Pontes A, Hutzler M, Brito PH, Sampaio JP (2020). Revisiting the Taxonomic Synonyms and Populations of Saccharomyces cerevisiae-Phylogeny, Phenotypes, Ecology and Domestication.. Microorganisms.

[R23] Preiss R, Tyrawa C, Krogerus K, Garshol LM, van der Merwe G (2018). Traditional Norwegian Kveik Are a Genetically Distinct Group of Domesticated Saccharomyces cerevisiae Brewing Yeasts.. Front Microbiol.

[R24] Raihofer L, Zarnow M, Gastl M, Hutzler M (2022). A short history of beer brewing: Alcoholic fermentation and yeast technology over time: Alcoholic fermentation and yeast technology over time.. EMBO Rep.

[R25] Štulíková Kateřina, Novák Jan, Vlček Jakub, Šavel Jan, Košin Petr, Dostálek Pavel (2020). Bottle Conditioning: Technology and Mechanisms Applied in Refermented Beers. Beverages.

[R26] Wei R, Wang L, Ding Y, Zhang L, Gao F, Chen N, Song Y, Li H, Wang H (2022). Natural and sustainable wine: a review.. Crit Rev Food Sci Nutr.

